# Renal function in patients with diabetic foot infection; does antibiotherapy affect it?

**DOI:** 10.15171/jrip.2017.23

**Published:** 2016-11-25

**Authors:** Roghayeh Akbari, Mostafa Javaniyan, Amir Fahimi, Mahmood Sadeghi

**Affiliations:** ^1^Clinical Research Development Unit of Ayatollah Rohani Hospital, Babol University of Medical Sciences, Babol, Iran; ^2^Infectious Diseases & Tropical Medicine Research Center, Babol University of Medical Sciences, Babol, Iran; ^3^Students Research Committee, Babol University of Medical Sciences, Babol, Iran

**Keywords:** Infection, Diabetic foot, Antibiotics, Glomerular filtration rate

## Abstract

**Introduction:** Antibiotic treatment (antibiotherapy) of diabetic foot ulcers has been proven to have toxic effect on renal function.

**Objectives:** This study aimed to evaluate renal function in patients with diabetic foot infection.

**Patients and Methods:** This cross-sectional retrospective study was performed on 142 patients with diabetic foot ulcers hospitalized in Shahid Yahyanejad hospital of Babol during 2013. After referring to profile of the patients, they were assigned to participate in two groups: group A consisted of patients receiving antibiotics with a low risk renal toxicity and patients who received antibiotics with a higher risk of renal toxicity were placed in group B. Glomerular filtration rate (GFR) was measured and calculated based on serum concentration of creatinine and Cockcroft-Gault equation. Data was analyzed using SPSS version 20.0 with chi-square, t test and paired t tests.

**Results:** Group A consisted of 74 patients (52.1%) and 68 patients (47.9%) participated in group B. GFRs before and after antibiotherapy were 64.73±33.87 cc/min and 59.10±30.51 cc/min, respectively (*P*=0.004). In group B, GFR decreased significantly after antibiotherapy (*P*=0.002).

**Conclusion:** According to the present study, renal function decreased after antibiotherapy and in patients who received antibiotics with higher nephrotoxicity rate, the rate of this decline was higher.

**Implication for health policy/practice/research/medical education:**

In a study on 74 patients (52.1%) receiving antibiotics with a lower risk of renal toxicity and 68 patients (47.9%) in the high-risk regimen, renal function before and after antibiotherapy were 64.73±33.87 cc/min and 59.10±30.51 cc/min, respectively (P=0.004). In group B, renal function significantly decreased after antibiotherapy (P=0.002). According to the present study, GFR decreased after antibiotherapy and in patients who received antibiotics with higher nephrotoxicity rate, the rate of this decline was higher.

## Introduction

Type 2 diabetes is one of the most common chronic diseases which causes major burdens on health system due to its increasing prevalence and vascular complications (1). It affects 285 million people around the world (2). Diabetic foot with subsequent infection is a frequent complication of diabetes both in developed and developing countries and one of the most common causes of morbidity and premature mortality in diabetics (3). In addition, it is expensive and estimated to affect 25% of all diabetic individuals during their lifetime due to neuropathy and potential coexisting vascular disease (4).

Diabetic foot process initiates with ulceration following a trauma that often goes unnoticed, mainly caused by diabetic neuropathy and incidentally by arteriopathy (5). Without a prompt and comprehensive assessment, it leads to ulceration and lower limb amputation frequently (6). The antibiotic treatment (antibiotherapy) plays an important role in therapy of these infections. Wounds without soft tissue or bone infection do not require antibiotic therapy. Empiric therapy covering gram positive cocci are used for treatment of mild and moderate infections whereas severe infections need broad spectrum gram-negative aerobes and obligate anaerobes (5).

The major side effect of aminoglycosides is nephrotoxicity which may occur in up to 20% of the patients (7). Also, at initial stages of diabetes, the kidneys grow large and the glomerular filtration rate (GFR) becomes greater than expected range (8). End-stage renal disease (ESRD) is an important factor in ulceration and increases major amputation for 2.5-3 times (9).

## Objectives

According to these issues and lack of information about diabetic patients in north of Iran and impact of different antibiotic regimens on renal function, we performed this study to evaluate GFR of these patients for assessment of renal function. The findings may be used for diagnostic and therapeutic decisions and choosing appropriate antibiotic regimen.

## Patients and Methods

This descriptive-retrospective study included 142 patients with diabetic foot hospitalized at Shahid-Yahyanejad hospital of Babol from March 2013 to 2014. Inclusion criteria were diagnosis of diabetes with presence of infected ulcers on a lower limb. Exclusion criteria were ESRD and amputation lower than knees. By referring to medical records of the patients, those who received antibiotics with a lower risk of renal toxicity (ciprofloxacin, ceftriaxone and clindamycin) participated in group A and patients who received antibiotics with a higher risk of renal toxicity (aminoglycosides, vancomycin and imipenem) participated in group B.

Patient information such as age, gender, location, diabetes duration, diabetes control method, body mass index (BMI), severity of the wound, creatinine before and after antibiotherapy, GFR and renal function before and after antibiotherapy were collected from patients’ records.

All foot were graded according to Wagner criteria. Grade 0 ulcers are pre- or post-ulcerative lesions, grade 1 ulcers are superficial, involving partial or full skin thickness, grade 2 ulcers are deeper, penetrating down to ligaments and joint capsule, and those of grade 3 are deep lesions, with abscesses or osteomyelitis (10). GFR was measured and calculated based on serum concentration of creatinine and the Cockcroft-Gault equation (11).

### Ethical issues

The research followed the tenets of the Declaration of Hel­sinki and was approved by the ethical committee of Babol University of Medical Sciences (#923478, 2013). Patients’ information remained confidential.

### Statistical analysis

The tests were performed at a significance level of 0.05. All data was analyzed with SPSS statistical software version 20.0 (SPSS Corp.), using chi-square, *t* test and paired *t* test. For descriptive statistical analysis, mean, standard deviations, absolute and relative frequencies were calculated. Statistical analysis was performed with the *t* test. To compare the variables between the groups, χ^2^ tests was performed for categorical data. Correlations between GFR and Wagner stages were done with the non-parametric Spearman coefficient test .

## Results

A total of 250 diabetic patients with diabetic foot were admitted and 142 cases were eligible for the study. The mean quantitative variables are shown in Table 1. Seventy-four patients (52.1%) were in low-risk group and 68 patients (47.9%) in the high-risk group. Most patients were female (n = 90, 63.4%), living in rural areas (n = 89, 62.7%), with glycemic control (n = 108, 76.1%), using oral glucose lowering medications (n = 96, 67.6%), Wagner stage 2 (n = 77, 54.2%) and Wagner stage 3 (n = 28, 19.7%).

**Table 1:** The mean quantitative variables of the study

**Variable**	**Mean±SD**	**Minimum**	**Maximum**
Age (year)	58.27±10.66	30	90
Diabetes duration (year)	14.49±7.16	8	40
Height (cm)	167.73±10.10	142	194
Weight (kg)	74.16±12.72	49	115
BMI (kg/m^2^)	26.97±3.90	18.86	34.45
Serum creatinine before antibiotherapy (mg/dL)	1.84±0.98	0.7	4.0
Serum creatinine after antibiotherapy (mg/dL)	1.80±1.08	0.8	5.1
GFR before antibiotherapy (cc/min)	64.73±33.87	15.00	120.00
GFR after antibiotherapy (cc/min)	59.10±30.51	15.00	120.00

Significant reduced renal function was observed in many variables which are shown in Table 2. The study variables were compared before and after antibiotherapy. We found that GFR decreased in both genders, but it was significant in females (63.79 ± 35.12 vs. 56.25 ± 31.76 cc/min, *P* = 0.003). Also, in patients with a very high BMI (obese people) decreased renal function was significant (69.85 ± 32.73 vs. 56.74 ± 24.76 cc/min, *P* = 0.004). In patients who have controlled their blood sugar with medication, renal function improved significantly after antibiotherapy (65.71 ± 34.37 vs. 53.80 ± 30.44 cc/min, *P* = 0.005). Based on Wagner category, renal function in the first three stages decreased significantly but its decrease was not significant in the last stage (*P* = 0.51).

**Table 2:** Renal function before and after antibiotherapy

**Variable**	**Renal function**		***P***
	**Before treatmentMean ± SD**	**After treatmentMean ± SD**	
Age group (years)			
<30	109.01 ± 0.00	104.13 ± 00	-
31-60	67.03 ± 35.01	63.41 ± 31.32	<0.001
61-90	60.67 ± 31.88	52.15 ± 27.91	<0.001
Gender			
Male	66.35 ± 31.86	64.03 ± 27.81	0.44
Female	63.79 ± 35.12	56.25 ± 31.76	0.003
Drug regimen			
Low risk	70.72 ± 31.77	60.07 ± 28.99	0.02
High risk	68.20 ± 35.10	50.04 ± 32.25	0.002
Diabetes duration (years)			
<10	66.64 ± 32.42	61.78 ± 29.45	0.34
11-20	63.20 ± 35.99	57.43 ± 32.86	0.08
21-30	58.49 ± 29.38	52.57 ± 25.67	0.18
31-40	72.04 ± 41.21	60.18 ± 29.92	0.09
Body mass index (kg/m^2^)			
Normal	58.97 ± 32.29	57.52 ± 29.33	0.59
Overweight	66.43 ± 35.60	61.70 ± 34.40	0.14
Obese	69.85 ± 32.73	56.74 ± 24.76	0.004
Glycemic control			
Nothing	61.73 ± 32.30	58.85 ± 29.96	0.47
oral agent	65.71 ± 34.37	53.80 ± 30.44	0.005
Insulin	65.37 ± 36.63	62.16 ± 34.93	0.57
Wound severity			
0	67.72 ± 38.01	55.57 ± 33.34	0.01
1	66.77 ± 38.59	54.82 ± 31.95	0.03
2	63.280 ± 32.45	59.87 ± 29.62	0.04
3	62.99 ± 33.74	65.10 ± 32.27	0.51

Renal function before and after antibiotherapy were 64.73 ± 33.87 and 59.10 ± 30.51 cc/min, respectively (*P* = 0.004). It decreased significantly in high-risk group (58.20 ± 35.10 vs. 50.04 ± 32.25 cc/min, *P* = 0.002). Although its decline was observed in the low-risk group, but it was not statistically significant (70.72 ± 31.77 vs. 65.07 ± 28.99 cc/min, *P* = 0.92; Table 3).

**Table 3:** Renal function based on the study groups

**Variable**	**Renal function**		**P**
	**Before treatment** **Mean±SD**	**After treatment** **Mean±SD**	
Group A (low risk regimen)	70.72 ± 31.77	65.07 ± 28.99	0.92
Group B (high risk regimen)	58.20 ± 35.10	50.04 ± 32.25	0.002

## Discussion

Foot ulcers and infections are one of the main causes of disability in patients with diabetes mellitus (12). About 15% of people with diabetes develop foot ulcers (13). Diabetic foot ulcers are frequently infected and takes a long time to heal. Today, in both developed and developing countries, diabetes is the most common cause of kidney problems (14). In more advanced stages of renal failure, when the GFR reaches less than 15-20 cc/min, renal clearance of insulin decreases. This is of more clinical importance in treatment of diabetes (15).

In this study, GFR decreased after antibiotherapy in diabetic patients. Wolf and Ritz noted that patients with diabetic foot infection are more vulnerable to impaired renal function (16). Lepäntalo et al believed that loss of renal function is considered to be an important factor for diabetic foot ulcers (9). Uremic toxins accumulation and parathyroid hormone level elevation of patients with chronic renal failure was shown to cause tissue insulin resistance, particularly in skeletal muscles which was mainly due to damaging insulin binding to receptors and cause disturbance of glucose metabolism and production of Glycogen (17). The study of Wolf et al in 2009 demonstrated a close association between renal function and diabetic foot. Also, a significant reverse relationship was found between GFR and Wagner stages of diabetic foot (18). Zubair and colleagues concluded that in diabetic patients, impaired renal function increases creatinine level and decreases GFR (19). Reduced GFR higher than normal range in people with diabetes tend to aggravate diabetic foot infection and ultimately leads to amputation (20). This is probably due to decreased insulin secretion in people with kidney disorders which might be due to metabolic acidosis, increased parathyroid hormone and decreased vitamin D level (21). Mamut et al, demonstrated that diabetes and GFR are significantly associated and independently affect renal function (22).

In this study, decreased renal function was found in patients receiving antibiotics. In patients who received antibiotics with higher renal toxicity, renal function decrease was significantly higher. Antibiotics are widely used in the treatment of infections. Their use can cause kidney damage. Also, most of the antibiotics are excreted through the kidneys (23). Pannell et al found that gentamicin has some side effects on kidney (24). However, regardless of the nature and mechanism, toxicity of antibiotics, depends on dose, concentration, duration, and other underlying diseases. In diabetic patients, antibiotherapy should be monitored carefully because renal dysfunction is more common in these patients and this trend is accelerated by the use of antibiotics (25). Unlike the therapeutic effects of aminoglycoside, its toxicity is not dose-dependent. Due to the high concentration of aminoglycosides in renal cortex and inner ear, nephrotoxicity is a common and important side effect. With increasing duration of treatment with these drugs, the risk of their nephrotoxicity increases, thus, if the treatment period exceeds 14 days, the risk of this complication reaches 50% (26). The final result of this process can be acute tubular necrosis and reduced GFR which is a life-threatening complication (27).

In this study, renal function reduced significantly in all age groups. However, older patients had more severe decline. It was obvious because the underlying disease increases with age and the organ function will deteriorate and the body’s ability to tolerate and take the waste out of the body decline.

## Conclusion

Based on the results of this study, renal function decreased after antibiotherapy. In patients receiving antibiotics with higher nephrotoxicity, its decline was higher. The results can be used to identify factors affecting renal function in patients with diabetic foot infections. Antibiotic prescription is not effective in prevention of diabetic foot infection and should be given in case of infection. In case of vancomycin administration, blood urea and creatinine should be checked regularly and if possible, simultaneous prescription of nephrotoxic drugs such as aminoglycosides should be avoided. Treatment of renal injury caused by aminoglycoside is basically support, discontinue of the drug, and substituting it for another non-nephrotoxic antibiotic. If it is not possible, the interval between aminoglycoside doses should be increase. Also, to maintain the balance of electrolytes and fluids, the use of other nephrotoxic drugs should be avoided (7).

## Limitations of the study

The limitation of this study was lack of antibiotic assessment separately. We had no incriminating data on social status of our patients and could not therefore rule out that the socioeconomic status may be a confounding factor in our study.

## Acknowledgments

It is our pleasure to thank all the laboratory staff of Shahid Yahyanejad hospital of Babol. We would also thank Mrs. Fatemeh Hosseinzadeh (Infectious Diseases Research Center with focus on Nosocomial Infections, Mazandaran University of Medical Sciences) for editing the manuscript.

## Authors’ contribution

RA and MS participated in all experiments, coordinat­ed the data-analysis and contributed to the writing of the manuscript. AF coordinated the acquisition of data. RA and MJ designed the research plan and organized the study. RA prepared the final manuscript.

## Conflicts of interests

The authors declare that they have no conflicts of interests.

## Ethical considerations

Ethical issues (including plagiarism, data fabrication, double publication) have been completely observed by the authors.

## Funding/Support

This article is extracted from general physician thesis of Amir Fahimi. This study was supported by a grant from Babol University of Medical Sciences (# 923649, 2013).
